# Analysis of the role of rs2031920 and rs3813867 polymorphisms within the *cytochrome P450 2E1* gene in the risk of squamous cell carcinoma

**DOI:** 10.1186/s12935-018-0561-8

**Published:** 2018-05-01

**Authors:** Hai Zhang, Haiyan Li, Huanxin Yu

**Affiliations:** Department of Otorhinolaryngology Head and Neck Surgery, Tianjin Huanhu Hospital, No 6, Ji Zhao Road, Jinnan District, Tianjin, 300060 People’s Republic of China

**Keywords:** *CYP2E1*, SCC, Polymorphism, Risk

## Abstract

**Background:**

To explore the genetic effect of rs2031920 and rs3813867 polymorphisms within the cytochrome P450 2E1 (*CYP2E1*) gene on the risk of squamous cell carcinoma (SCC), a meta-analysis was performed.

**Methods:**

The eligible case–control studies were obtained by database searching and screening, and the specific statistical analysis was performed with STATA 12.0 software.

**Results:**

After the process of database searching and screening, a total of 32 case–control studies with 7435 cases and 10,466 controls were ultimately included in our meta-analysis. With regard to the rs2031920 C/T polymorphism, in comparison to controls, a reduced risk in cases of esophageal squamous cell carcinoma (ESCC) was detected for the models of allele T vs. allele C [*P *= 0.025, odds ratio (OR) = 0.67], carrier T vs. carrier C (*P *= 0.014, OR = 0.70), TT vs. CC (*P *= 0.029, OR = 0.65), CT vs. CC (*P *= 0.040, OR = 0.56), CT + TT vs. CC (*P *= 0.035, OR = 0.58). Similarly, a decreased SCC risk was observed for the rs3813867 G/C polymorphism in the allele, carrier, homozygote, dominant, and recessive models of overall SCC meta-analysis and “ESCC” subgroup analysis (all *P *< 0.05, OR < 1) and in all genetic models of “Asian” and “population-based control (PB)” subgroup analysis (all *P *< 0.05, OR < 1). Additionally, for the rs2031920/rs3813867 haplotype, a decreased SCC risk was also detected in the overall SCC meta-analysis under the allele, carrier, homozygote and dominant model (all *P *< 0.05, OR < 1) and the subgroup analysis of “PB” under the allele, carrier, and dominant models (all *P *< 0.05, OR < 1).

**Conclusions:**

Our meta-analysis supports the “T” allele carrier of the C*YP2E1* rs2031920 C/T polymorphism and “C” allele carrier of the rs3813867 G/C polymorphism as protective factors for ESCC patients, especially in Asian populations.

## Background

The cytochrome P450 2E1 (*CYP2E1*) gene in *Homo sapiens* is located on chromosome 10 and is responsible for encoding a membrane-bound CYP2E1 protein, an important member of the human cytochrome P450 system [1]. The cytochrome P450 system works as a series of phase I enzymes participating in a group of biological events, such as drug metabolism, oxidative reactions, or the detoxification of endogenous and exogenous substances [2, 3]. Polymorphic variants, existing in the functional genes of the cytochrome P450 system, are associated with the pathogenesis of several clinical cancers [2, 3]. For example, rs2031920 C/T with an *Rsa*I restriction enzyme site and rs3813867 C/T with a *Pst*I restriction enzyme site are two common single nucleotide polymorphisms (SNP) within the 5′-flanking regions of the *CYP2E1* gene [4–6]. Three genotypes of c1/c1, c1/c2, c2/c2 were generated; rs2031920 and rs3813867 were in close linkage disequilibrium [4–6]. Furthermore, *CYP2E1* polymorphisms were reported to be linked to several cancers, such as nasopharyngeal carcinoma [7], urinary cancers [6] and head and neck carcinoma [5], particularly in Asian populations.

Squamous cell carcinoma (SCC) is the most common histological type of several clinical cancers, such as head and neck cancer, esophageal cancer, skin cancer, lung cancer, and cervical cancer [8, 9]. The exact pathogenesis of SCC remains unclear. Living habits (e.g., smoking, drinking), viral infection [e.g., human papillomavirus (HPV)], immune system, and polymorphic variants with many genes may be related to the risk of different SCC diseases [10–12]. Previously, we conducted an updated meta-analysis to explore the impact of *MDM2* (MDM2 Proto-Oncogene) polymorphisms on SCC susceptibility and found that the GG genotype of *MDM2* rs2279744 polymorphism may be associated with an increased risk of esophageal SCC in Asian populations [8].

We observed a different conclusion regarding the role of rs2031920 and rs3813867 polymorphisms within the *CYP2E1* gene in the risk of SCC. Thus, we are very interested in investigating the role of the rs2031920 and rs3813867 polymorphisms within the *CYP2E1* gene in the susceptibility to SCC, considering the lack of publications of specific meta-analyses. We included a total of 32 case–control studies in our meta-analysis, which followed the preferred reporting items for systematic reviews and meta-analyses (PRISMA) [13].

## Methods

### Database searching and screening

Five electronic databases, including PubMed, Web of Science, Cochrane, Scopus and Chinese National Knowledge Infrastructure (CNKI), were searched prior to January 2018. We used a group of keywords: Carcinoma, Squamous Cell; Carcinomas, Squamous Cell; Squamous Cell Carcinomas; Squamous Cell Carcinoma; Carcinoma, Squamous; Carcinomas, Squamous; Squamous Carcinoma; Squamous Carcinomas; Carcinoma, Epidermoid; Carcinomas, Epidermoid; Epidermoid Carcinoma; Epidermoid Carcinomas; Carcinoma, Planocellular; Carcinomas, Planocellular; Planocellular Carcinoma; Planocellular Carcinomas; esophageal squamous cell carcinoma head and neck squamous cell carcinoma; lung squamous cell carcinoma; skin squamous cell carcinoma; oral squamous cell carcinoma; cervix squamous cell carcinoma; vagina squamous cell carcinoma; SCC; ESCC; HNSCC; LSCC; SSCC; OSCC; Cytochrome P-450 CYP2E1; Cytochrome P 450 CYP2E1; Cytochrome P-450-J; Cytochrome P 450 J; 4-Nitrophenol-2-Hydroxylase; 4 Nitrophenol 2 Hydroxylase; Dimethylnitrosamine *N*-Demethylase; Dimethylnitrosamine *N* Demethylase; Cytochrome P450 2E1; *N*-Nitrosodimethylamine Demethylase; *N* Nitrosodimethylamine Demethylase; CYP2E1; Cytochrome P-450 IIE1; Cytochrome P 450 IIE1; CYP IIE1; CYPIIE1; Cytochrome P-450 (ALC).

The retrieved studies were then reviewed and screened with the following exclusion criteria: (1) data based on animal experiments; (2) case reports, cohort studies or meeting abstracts; (3) without SNP data; (4) meta-analyses or reviews; (5) no SCC or CYP2E1 data; (6) duplicate studies; (7) no pathological typing data; (8) no genotype data. The data of genotype frequencies in cases and controls must have been provided in the selected studies.

### Characteristics and quality assessment

Based on the eligible articles, the authors extracted and summarized the usable information, including the first author’s name, year, country, race, SNP, genotype frequency, SCC type, control source, genotyping assay, and HWE (Hardy–Weinberg equilibrium), in a table. The Newcastle–Ottawa Scale (NOS) system was also used to assess the methodological quality of individual studies. Only the studies with NOS score > 5 were ultimately included in our meta-analysis.

### Heterogeneity and association test

STATA software (Stata Corporation, College Station, TX, USA) was used for our heterogeneity and association tests. In the case of heterogeneity, the *P* value of Cochran’s Q statistic < 0.05 or I^2^ value > 50% were considered to represent high heterogeneity among studies, which led to the use of a random effects model (DerSimonian and Laird method). Otherwise, the fixed effects model (Mantel–Haenszel statistics) was used. In the association test, odds ratio (OR), 95% confidence interval (CI) and *P* value were computed to assess the association strength in the allele, carrier, homozygote, heterozygote, dominant, and recessive models. In addition, based on the factors of race, SCC type, control source and HWE, a series of subgroup analyses were performed as well.

### Publication bias and sensitivity analysis

Begg’s test and Egger’s test were used to assess the potential publication bias. A *P* value larger than 0.05 indicated the absence of potential publication bias. In addition, sensitivity analysis was used to evaluate the data stability and possible sources of heterogeneity.

## Results

### Process for identifying eligible studies

**Fig. 1:**
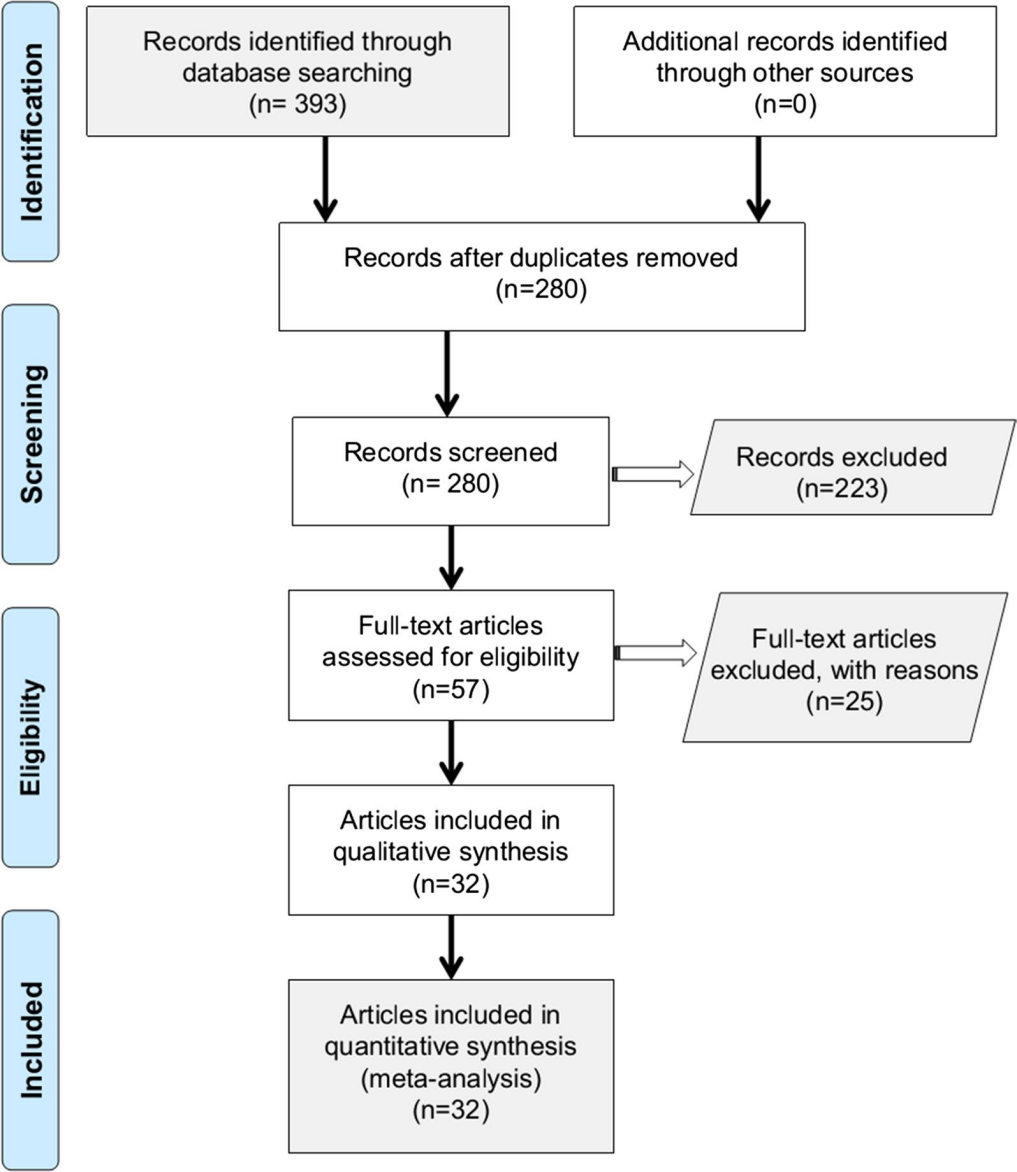
The process for identifying eligible studies

After our initial database retrieval, a total of 393 records [PubMed (n = 89), Web of Science (n = 161), Cochrane (n = 1), Scopus (n = 116) and CNKI (n = 26)] were obtained, as presented in Fig. 1. Then, 113 duplicate records were excluded. Based on the exclusion criteria, 223 records were removed. Moreover, the lack of confirmed pathological typing data or genotype frequency distribution resulted in the exclusion of another 25 articles. Finally, our meta-analysis involved a total of 32 articles [14–45] containing 7435 cases and 10,466 controls. The characteristics of each study are presented in Table 1. No study had poor quality; the NOS score of all studies was greater than five (Table 1).

**Table 1:** Characteristics of each study included in the meta-analysis

First author	Year	Country	Race	NOS	SNP	Case		Assay	Control		
						AA/AB/BB	Type		AA/AB/BB	Source	HWE
Balaji	2011	India	Mixed	8	rs3813867	151/6/0	HNSCC	TaqMan allelic discrimination	125/7/0	PB	Y
					rs2031920	151/6/0	HNSCC	TaqMan allelic discrimination	125/7/0	PB	Y
					rs2031920/rs3813867	151/6/0	HNSCC	TaqMan allelic discrimination	125/7/0	PB	Y
Bhat	2014	India	Asian	6	rs2031920	366/148/12	ESCC	PCR–RFLP	207/308/11	HB	N
Bouchardy	2000	France	Caucasian	7	rs2031920	109/11/1	HNSCC	PCR–RFLP	164/8/0	HB	Y
Brocic	2011	Serbia	Caucasian	9	rs2031920	105/13/5	HNSCC	PCR–RFLP	160/16/1	PB	Y
Cao	2014	China	Asian	7	rs3813867	143/44/2	LSCC	PCR–RFLP	340/168/18	HB	Y
Cury	2012	Brazil	Africa	7	rs3813867	160/14^1^	HNSCC	PCR–RFLP	242/36^1^	PB	Y
Ferreira	2006	Portugal	Caucasian	7	rs2031920	113/9^1^	CSCC	PCR–RFLP	224/11^1^	PB	Y
Gajecka	2005	Poland	Caucasian	6	rs2031920	279/9/0	HNSCC	PCR–RFLP	305/18/0	PB	Y
Gattas	2006	Brazil	Africa	6	rs3813867	90/13/0	HNSCC	PCR–RFLP	96/6/0	HB	Y
					rs3813867	31/7/0^a^	HNSCC	PCR–RFLP	96/6/0	HB	Y
Gonzalez	1998	Spain	Caucasian	6	rs3813867	68/6/1	HNSCC	PCR–RFLP	179/21/0	PB	Y
Guo	2012	China	Asian	8	rs2031920	195/125^2^	HNSCC	PCR–RFLP	254/66^2^	PB	NR
Guo	2008	China	Asian	8	rs2031920/rs3813867	57/16/7	ESCC	PCR–RFLP	225/180/75	PB	N
Huang	2000	China	Asian	8	rs2031920	10/13/1	LSCC	PCR–RFLP	152/101/7	PB	N
Le	1998	USA	Mixed	8	rs2031920	56/17/1	LSCC	PCR–RFLP	338/102/14	PB	Y
Lee	2006	Korea	Asian	6	rs2031920	30/37/6	LSCC	PCR–RFLP	90/89/12	HB	Y
Li, D	2005	South Africa	Mixed	8	rs2031920	184/5/0	ESCC	SSCP	191/7/0	PB	Y
					rs3813867	184/5/0	ESCC	SSCP	187/11/0	PB	Y
Li	2008	China	Asian	7	rs2031920/rs3813867	39/31^1^	LSCC	PCR–RFLP	83/69^1^	PB	Y
Li, G	2005	USA	Caucasian	6	rs3813867	684/37/3	HNSCC	PCR–RFLP	1137/86/3	HB	Y
Li	2011	China	Asian	6	rs3813867	159/67/0	ESCC	PCR–RFLP	173/62/11	HB	Y
Li	2000	China	Asian	7	rs2031920	40/11/2	LSCC	PCR–RFLP	75/57/5	PB	Y
Liu	2007	China	Asian	8	rs2031920	34/33/10	ESCC	PCR–RFLP	45/29/5	PB	Y
Matthias	2003	Germany	Caucasian	6	rs2031920/rs3813867	307/18/1^b^	HNSCC	PCR–RFLP	165/10/0	HB	Y
					rs2031920/rs3813867	35/3/0^c^	HNSCC	PCR–RFLP	165/10/0	HB	Y
Morita	1997	Japan	Asian	8	rs2031920/rs3813867	34/18/1	ESCC	PCR–RFLP	85/42/5	PB	Y
Neuhaus	2004	Germany	Caucasian	6	rs2031920	304/8/0	HNSCC	PCR	282/13/2	PB	N
Nishino	2008	Japan	Asian	6	rs2031920	74/44/6	CSCC	PCR–RFLP	68/42/7	PB	Y
Oyama	2002	Japan	Asian	6	rs2031920	40/8/5	LSCC	PCR–RFLP	391/196/25	PB	Y
Pandey	2012	India	Caucasian	7	rs2031920	47/3^1^	HNSCC	PCR–RFLP	35/15^1^	PB	NR
Ruwali	2010	India	Asian	6	rs2031920	327/23^1^	HNSCC	NR	343/7^1^	PB	NR
Soya	2008	India	Asian	7	rs2031920/rs3813867	394/14^1^	HNSCC	PCR–RFLP	212/8^1^	HB	Y
Tai	2010	China	Asian	9	rs2031920	184/81/13	HNSCC	PCR–RFLP	182/84/12	PB	Y
Tan	2010	China	Asian	9	rs2031920	107/31/12	ESCC	PCR–RFLP	66/77/7	PB	N
Wang	2012	China	Asian	8	rs3813867	156/74/10^d^	ESCC	Gel-based DNA microarray	131/94/20	PB	Y
					rs3813867	149/85/8^e^	ESCC	Gel-based DNA microarray	109/94/18	PB	Y
					rs2031920	154/76/10^d^	ESCC	Gel-based DNA microarray	131/94/20	PB	Y
					rs2031920	141/93/8^e^	ESCC	Gel-based DNA microarray	108/95/18	PB	Y

*SNP* single nucleotide polymorphisms, *NOS* Newcastle–Ottawa Scale*, HNSCC* head and neck squamous cell carcinoma, *ESCC* esophageal squamous cell carcinoma; *LSCC* lung squamous cell carcinoma, *CSCC* cervical squamous cell carcinoma, *A* major allele, *B* minor allele, *PCR* polymerase chain reaction, *RFLP* restriction fragment-length polymorphism, *SSCP* single-strand conformation polymorphism, *NR* not reported, *PB* population-based control, *HB* hospital-based control, *HWE* hardy–weinberg equilibrium, *Y P* value of HWE > 0.05, *N P* value of HWE > 0.05

^1^ The genotype frequencies of “AA/AB + BB”

^2^ The genotype frequencies of “AA + AB/BB”

^a^ Data of oral squamous cell carcinoma

^b^ Single HNSCC

^c^ Multiple HNSCC

^d^ Data from “Chaoshan” region

^e^ Data from “Taihang” region

### The rs2031920 polymorphism

A meta-analysis of rs2031920 and SCC risk was conducted on the allele model (allele T vs. allele C), carrier model (carrier T vs. carrier C), homozygote model (TT vs. CC), heterozygote model (CT vs. CC), dominant model (CT + TT vs. CC), and recessive model (TT vs. CC + CT). As shown in Table 2, 18 case–control studies were enrolled for the allele, carrier, heterozygote models, 15 case–control studies were enrolled for the homozygote model, 21 case–control studies were enrolled for the dominant model, and 16 case–control studies were enrolled for the recessive model. Pooling results suggested that there was no statistically significant difference for the overall SCC risk between the case and control groups under any model (Table 2, *P* value of association test > 0.05).

**Table 2:** Meta-analysis of *CYP2E1* rs2031920 C/T polymorphism and SCC risk

Comparisons	Group	Number (study)	OR	95% CI	*P* (association)
Allele model (allele T vs. allele C)	All	18	0.84	0.67–1.06	0.144
	Asian	11	0.80	0.61–1.05	0.106
	Caucasian	4	1.04	0.46–2.37	0.929
	HNSCC	6	0.99	0.62–1.59	0.971
	ESCC	6	0.67	0.48–0.95	0.025
	LSCC	5	0.94	0.67–1.32	0.722
	PB	15	0.83	0.68–1.02	0.076
	HB	3	1.00	0.38–2.58	0.994
	Y	14	0.92	0.75–1.13	0.449
	N	4	0.60	0.37–0.99	0.048
Carrier model (carrier T vs. carrier C)	All	18	0.83	0.69–1.01	0.064
	Asian	11	0.80	0.60–1.00	0.053
	Caucasian	4	0.99	0.49–1.99	0.982
	HNSCC	6	0.96	0.65–1.43	0.849
	ESCC	6	0.70	0.53–0.93	0.014
	LSCC	5	0.92	0.68–1.25	0.602
	PB	15	0.83	0.71–0.98	0.027
	HB	3	0.98	0.44–2.16	0.955
	Y	14	0.91	0.78–1.06	0.236
	N	4	0.62	0.42–0.92	0.018
Homozygote model (TT vs. CC)	All	15	0.87	0.65–1.15	0.324
	Asian	11	0.83	0.62–1.12	0.324
	Caucasian	3	2.18	0.66–7.19	0.202
	HNSCC	4	1.35	0.69–2.62	0.379
	ESCC	5	0.65	0.44–0.96	0.029
	LSCC	5	1.27	0.69–2.33	0.440
	PB	12	0.85	0.62–1.17	0.316
	HB	3	0.94	0.49–1.79	0.847
	Y	11	0.90	0.65–1.24	0.522
	N	4	0.76	0.42–1.38	0.371
Heterozygote model (CT vs. CC)	All	18	0.74	0.54–1.02	0.067
	Asian	11	0.68	0.45–1.02	0.064
	Caucasian	4	0.93	0.51–1.71	0.825
	HNSCC	6	0.92	0.66–1.28	0.617
	ESCC	6	0.56	0.32–0.97	0.040
	LSCC	5	0.82	0.45–1.47	0.503
	PB	15	0.73	0.56–0.96	0.024
	HB	3	0.85	0.23–3.17	0.804
	Y	14	0.85	0.69–1.05	0.139
	N	4	0.48	0.23–1.01	0.054
Dominant model (CT + TT vs. CC)	All	21	0.81	0.60–1.11	0.189
	Asian	12	0.80	0.54–1.19	0.263
	Caucasian	6	0.85	0.42–1.71	0.644
	HNSCC	8	0.95	0.56–1.62	0.844
	ESCC	6	0.58	0.35–0.96	0.035
	LSCC	5	0.87	0.53–1.44	0.591
	PB	18	0.81	0.61–1.07	0.138
	HB	3	0.89	0.24–3.33	0.864
	Y	15	0.90	0.72–1.12	0.345
	N	4	0.50	0.25–0.99	0.046
Recessive model (TT vs. CC + CT)	All	16	1.21	0.80–1.83	0.362
	Asian	12	1.20	0.78–1.84	0.402
	Caucasian	3	2.11	0.23–19.71	0.512
	HNSCC	5	1.88	0.91–3.90	0.089
	ESCC	5	0.91	0.47–1.74	0.770
	LSCC	5	1.47	0.81–2.69	0.206
	PB	13	1.18	0.72–1.94	0.514
	HB	3	1.24	0.66–2.34	0.497
	Y	11	1.05	0.65–1.71	0.829
	N	4	1.26	0.70–2.28	0.438

*OR* odds ratio, *CI* confidence interval, *HNSCC* head and neck squamous cell carcinoma, *ESCC* esophageal squamous cell carcinoma, *LSCC* lung squamous cell carcinoma, *PB* population-based control, *HB* hospital-based control, *Y P* value of hardy–weinberg equilibrium > 0.05, *N P* value of hardy–weinberg equilibrium > 0.05

**Fig. 2:**
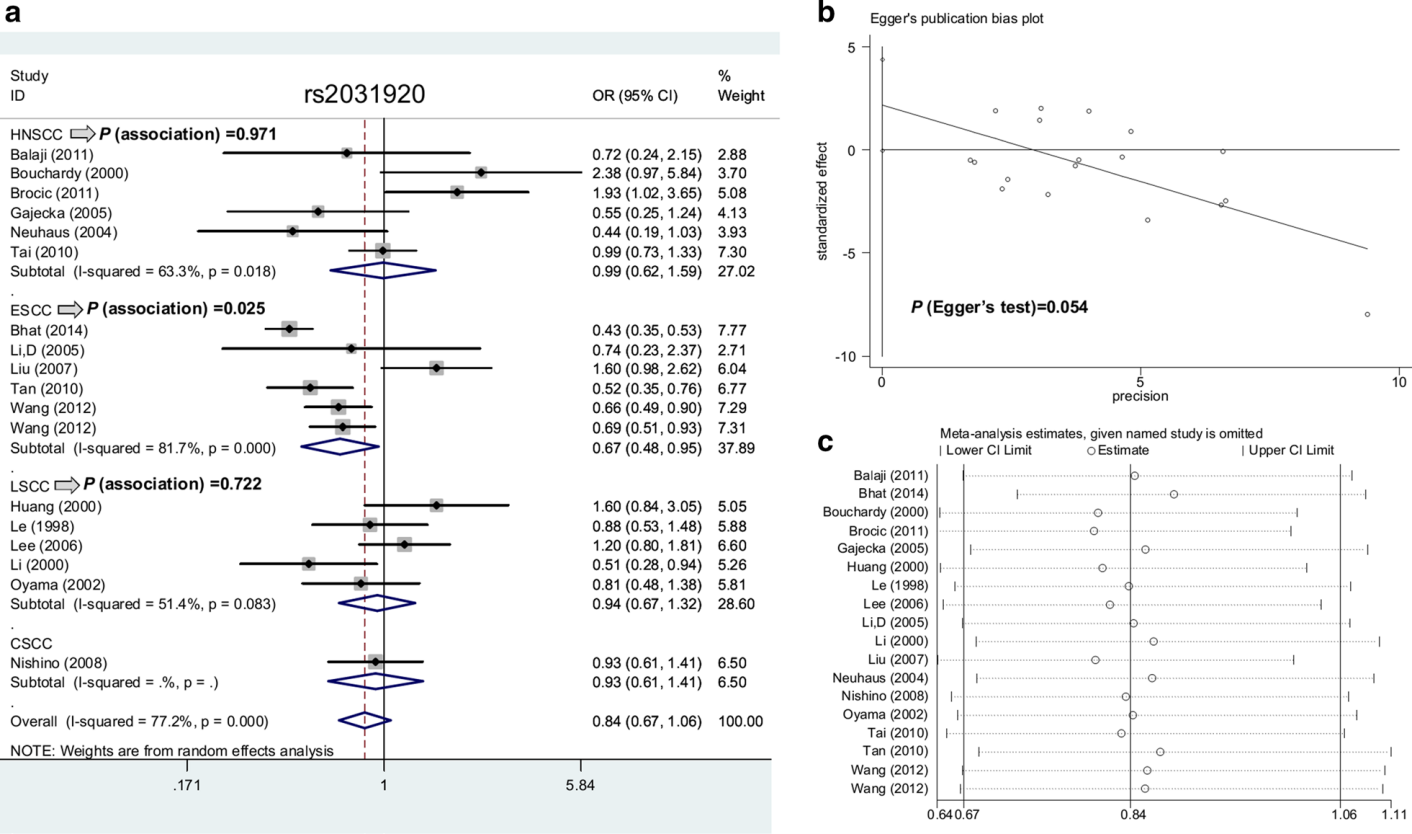
Meta-analysis data of rs2031920 under the allele model. **a** Subgroup analysis according to the SCC type; **b** Egger’s test; **c** sensitivity analysis

Moreover, we conducted a statistical analysis of the subgroup of race (Asian/Caucasian), SCC type (HNSCC/ESCC/LSCC), control source (PB/HB), and HWE (Y/N). As shown in Table 2, in comparison with controls, a reduced ESCC risk was observed in the models of allele T vs. allele C (*P *= 0.025, OR = 0.67), carrier T vs. carrier C (*P *= 0.014, OR = 0.70), TT vs. CC (*P *= 0.029, OR = 0.65), CT vs. CC (*P *= 0.040, OR = 0.56), CT + TT vs. CC (*P *= 0.035, OR = 0.58), but not TT vs. CC + CT (*P *= 0.770). Figure 2a shows forest plot data in subgroup analysis by SCC type under the allele model. The “T” allele carrier of the rs2031920 polymorphism within the *CYP2E1* gene seems to be linked to ESCC risk.

### The rs3813867 polymorphism

**Fig. 3:**
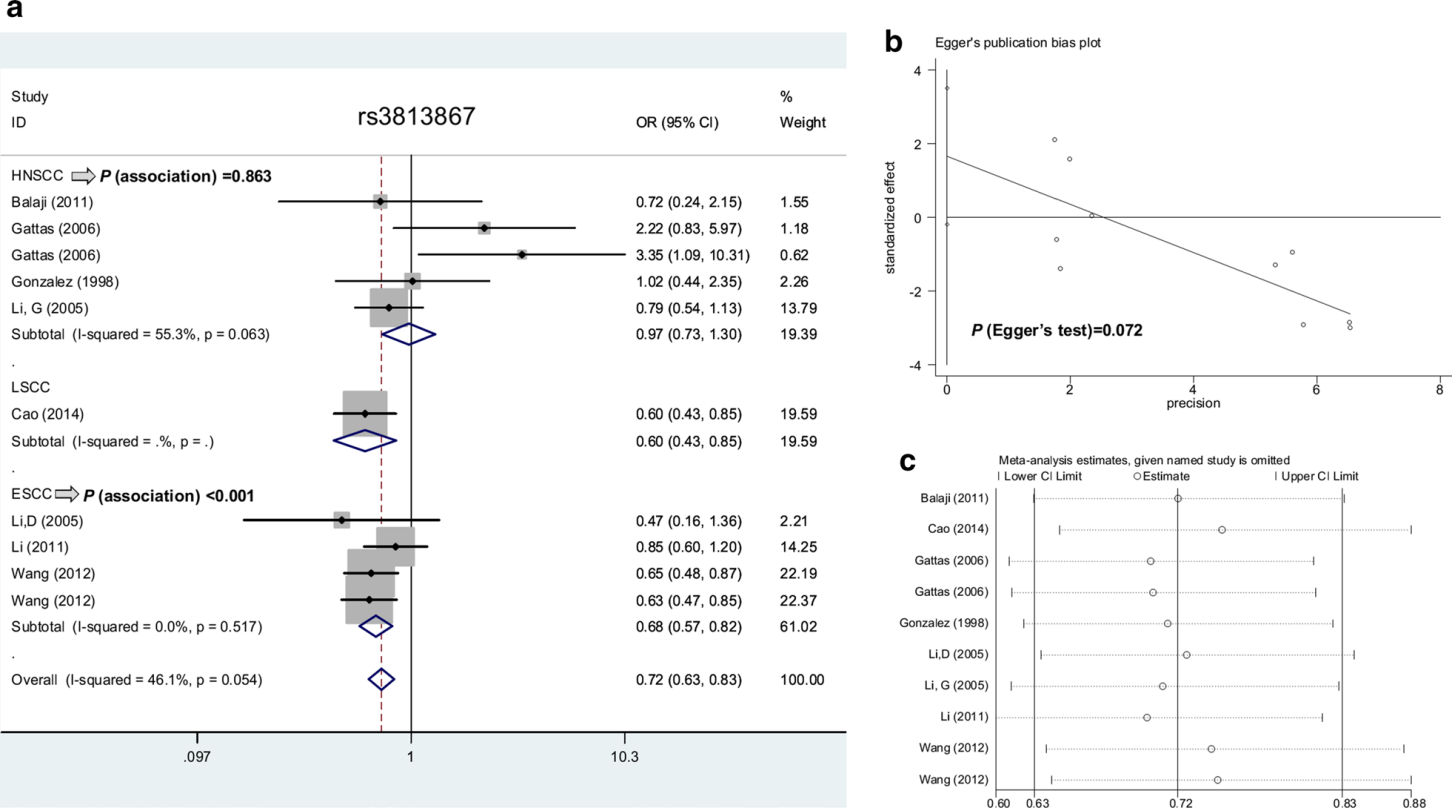
Meta-analysis data of rs3813867 under the allele model. **a** Subgroup analysis according to the SCC type; **b** Egger’s test; **c** sensitivity analysis

We also conducted the overall and subgroup meta-analysis of rs3813867 and SCC risk under the allele (10 case–control studies), carrier (10 case–control studies), homozygote (6 case–control studies), heterozygote (10 case–control studies), dominant (11 case–control studies), and recessive (6 case–control studies) models. The positive results regarding the association between *CYP2E1* rs3813867 and SCC risk were detected in the overall SCC meta-analysis and subgroup analysis of “ESCC” and “Y” (*P* value of Hardy–Weinberg equilibrium > 0.05) under all genetic models (Table 3, all *P *< 0.05, OR < 1), only apart from the heterozygote model (*P *= 0.150). A decreased SCC risk was also detected in the subgroup analysis of “Asian” and “PB” under all genetic models (Table 3, all *P *< 0.05, OR < 1). Figure 3a shows the forest plot data of subgroup analysis by SCC type under the allele model. The “C” allele carrier of *CYP2E1* rs3813867 polymorphism may be associated with the risk of SCC, especially the ESCC cases in Asian populations.

**Table 3:** Meta-analysis of *CYP2E1* rs3813867 G/C polymorphism and SCC risk

Comparisons	Group	Number (study)	OR	95% CI	*P* (association)
Allele model (allele C vs. allele G)	All	10	0.72	0.63–0.83	< 0.001
	Asian	4	0.67	0.57–0.78	< 0.001
	HNSCC	5	0.97	0.73–1.30	0.863
	ESCC	4	0.68	0.57–0.82	< 0.001
	PB	5	0.65	0.53–0.79	< 0.001
	HB	5	0.80	0.66–0.97	0.021
	Y	10	0.72	0.63–0.83	< 0.001
Carrier model (carrier C vs. carrier G)	All	10	0.79	0.68–0.92	0.002
	Asian	4	0.75	0.63–0.90	0.001
	HNSCC	5	0.94	0.70–1.27	0.698
	ESCC	4	0.77	0.63–0.93	0.008
	PB	5	0.72	0.58–0.89	0.003
	HB	5	0.85	0.70–1.05	0.133
	Y	10	0.79	0.68–0.92	0.002
Homozygote model (CC vs. GG)	All	6	0.38	0.24–0.61	< 0.001
	Asian	4	0.30	0.18–0.50	< 0.001
	HNSCC	2	2.34	0.58–9.34	0.230
	ESCC	3	0.30	0.17–0.53	< 0.001
	PB	3	0.43	0.24–0.75	0.003
	HB	3	0.30	0.12–0.74	0.009
	Y	6	0.38	0.24–0.61	< 0.001
Heterozygote model (GC vs. GG)	All	10	0.82	0.63–107	0.150
	Asian	4	0.75	0.56–0.99	0.045
	HNSCC	5	1.15	0.62–2.16	0.657
	ESCC	4	0.76	0.54–1.07	0.116
	PB	5	0.66	0.51–0.84	0.001
	HB	5	1.09	0.67–1.76	0.730
	Y	10	0.82	0.63–107	0.150
Dominant model (GC + CC vs. GG)	All	11	0.76	0.60–0.97	0.024
	Asian	4	0.68	0.54–0.86	0.002
	HNSCC	6	1.01	0.62–1.65	0.961
	ESCC	4	0.70	0.53–0.92	0.011
	PB	6	0.62	0.50–0.77	< 0.001
	HB	5	1.03	0.65–1.62	0.916
	Y	11	0.76	0.60–0.97	0.024
Recessive model (CC vs. GG + GC)	All	6	0.43	0.27–0.68	< 0.001
	Asian	4	0.34	0.20–0.57	< 0.001
	HNSCC	2	2.39	0.60–9.53	0.218
	ESCC	3	0.35	0.20–0.60	< 0.001
	PB	3	0.49	0.28–0.86	0.013
	HB	3	0.31	0.13–0.77	0.011
	Y	6	0.43	0.27–0.68	< 0.001

*OR* odds ratio, *CI* confidence interval, *HNSCC* head and neck squamous cell carcinoma, *ESCC* esophageal squamous cell carcinoma, *PB* population-based control, *HB* hospital-based control, *Y P* value of hardy–weinberg equilibrium > 0.05

### The rs2031920/rs3813867 haplotype

The results of overall and subgroup meta-analysis of the rs2031920/rs3813867 haplotype and SCC risk under the allele (five case–control studies), carrier (five studies), homozygote (three studies), heterozygote (five studies), dominant (seven studies), and recessive (three studies) models are shown in Table 4. We observed a decreased SCC risk in the overall SCC meta-analysis under the allele, carrier, homozygote, and dominant models (Table 4, all *P *< 0.05, OR < 1), and the subgroup analysis of “PB” under the allele, carrier, and dominant models (all *P *< 0.05, OR < 1). These results suggested a potential link between the c1/c2 or c2/c2 of rs2031920/rs3813867 haplotype and SCC risk, which still requires more case–control studies.

**Table 4:** Meta-analysis of *CYP2E1* rs2031920/rs3813867 haplotype and SCC risk

Comparisons	Group	Number (study)	OR	95% CI	*P* (association)
Allele c2 vs. allele c1	All	5	0.65	0.49–0.86	0.003
	HNSCC	3	1.01	0.57–1.78	0.977
	PB	3	0.57	0.42–0.79	0.001
	Y	4	0.98	0.65–1.46	0.913
Carrier c2 vs. carrier c1	All	5	0.73	0.53–1.00	0.047
	HNSCC	3	0.98	0.55–1.75	0.945
	PB	3	0.65	0.45–0.93	0.019
	Y	4	0.98	0.64–1.50	0.938
c2c2 vs. c1c1	All	3	0.41	0.20–0.86	0.018
c1c2 vs. c1c1	All	5	0.75	0.43–1.30	0.309
	HNSCC	3	0.96	0.53–1.71	0.877
	PB	3	0.63	0.29–1.35	0.231
	Y	4	1.00	0.64–1.56	0.990
c1c2 + c2c2 vs. c1c1	All	7	0.72	0.55–0.94	0.016
	HNSCC	4	0.97	0.59–1.57	0.892
	PB	4	0.64	0.47–0.87	0.005
	Y	6	0.97	0.70–1.35	0.871
c2c2 vs. c1c1 + c1c2	All	3	0.55	0.26–1.13	0.103

*OR* odds ratio, *CI* confidence interval, *HNSCC* head and neck squamous cell carcinoma, *PB* population-based control, *Y P* value of hardy–weinberg equilibrium > 0.05

### Heterogeneity evaluation

When assessing the heterogeneity level, the fixed model was used for the TT vs. CC model of rs2031920 due to the lack of high heterogeneity (Table 5, I^2^ = 38.3%, *P* value of heterogeneity = 0.066), however, the random model was utilized for others. The fixed model was used for the allele, carrier, homozygote and recessive models of rs3813867 (Table 5, all I^2^ < 50.0%, *P* value of heterogeneity > 0.05); and the allele, carrier, homozygote, dominant, and recessive models of the rs2031920/rs3813867 haplotype (Table 5, all I^2^ < 50.0%, *P* value of heterogeneity > 0.05).

**Table 5:** Heterogeneity test and publication analysis

SNP	Comparisons	I^2^ (%)	*P* (heterogeneity)	F/R	*P* (Begg’s test)	*P* (Egger’s test)
rs2031920 (C/T)	Allele T vs. allele C	77.2	< 0.001	R	0.649	0.054
	Carrier T vs. carrier C	58.9	0.001	R	0.449	0.077
	TT vs. CC	38.3	0.066	F	0.276	0.242
	CT vs. CC	82.1	< 0.001	R	0.544	0.544
	CT + TT vs. CC	83.1	< 0.001	R	0.608	0.037
	TT vs. CC + CT	57.4	0.002	R	0.685	0.207
rs3813867 (G/C)	Allele C vs. allele G	46.1	0.054	F	0.074	0.072
	Carrier C vs. carrier G	28.4	0.183	F	0.107	0.150
	CC vs. GG	45.4	0.103	F	0.707	0.651
	GC vs. GG	52.4	0.026	R	0.107	0.230
	GC + CC vs. GG	47.3	0.041	R	0.062	0.150
	CC vs. GG + GC	43.6	0.115	F	1.000	0.732
rs2031920 + rs3813867 (c1/c2)	Allele c2 vs. allele c1	49.8	0.093	F	1.000	0.184
	Carrier c2 vs. carrier c1	15.5	0.316	F	0.806	0.245
	c2c2 vs. c1c1	0.0	0.671	F	0.296	0.269
	c1c2 vs. c1c1	53.1	0.074	R	0.806	0.327
	c1c2 + c2c2 vs. c1c1	46.3	0.083	F	0.764	0.227
	c2c2 vs. c1c1 + c1c2	0.0	0.792	F	0.296	0.501

*SNP* single nucleotide polymorphisms, *F* fixed, *R* random

### Publication bias and sensitivity analysis

Begg’s and Egger’s tests did not provide confirmed evidence of obvious publication bias in the above comparisons (Table 5, all *P* value of Begg’s test and Egger’s test> 0.05) apart from the CT + TT vs. CC model of rs2031920 (*P* value of Egger’s test = 0.037). Figures 2b and 3b show the Egger’s publication bias plot of rs2031920 and rs3813867 under the allele model, respectively. Additionally, a relatively stable conclusion was obtained by sensitivity analysis results (Fig. 2c for allele model of rs2031920; Fig. 3c for allele model of rs3813867; data for others not shown).

## Discussion

*CYP2E1* rs2031920 was related to the risk of ESCC in a high-incidence region (Kashmir, India) [15]. Nevertheless, negative results were also reported in one study from South Africa [29] and in a Huai’an population from China [34]. Meta-analysis can address this conflicting issue. We did not observe published meta-analyses specific for the genetic relationship between *CYP2E1* rs2031920, rs3813867 SNP and ESCC risk. In this study, we provide evidence that the “T” allele carrier of the rs2031920 polymorphism and the “C” allele carrier of the *CYP2E1* rs3813867 polymorphism may be associated with a decreased risk of ESCC, especially in Asian populations because most of the included case–control studies were from China or India.

Tang et al. [46] selected 21 case–control studies for a meta-analysis in 2010 and investigated the potential effect of *CYP2E1* rs2031920 and rs3813867 in the risk of head and neck cancer; they found that the homozygote genotype of *CYP2E1* rs2031920/rs3813867 may be linked to the risk of head and neck cancer, especially in Asian populations. Zhuo et al. [5] performed another meta-analysis containing 43 case–control studies in 2016 and reported a positive association between *CYP2E1* rs2031920/rs3813867 and head and neck cancer risk under the homozygote model. However, the subgroup analysis based of HNSCC was not performed in the two meta-analyses. In our meta-analysis, we failed to observe the statistical relationship between *CYP2E1* rs2031920 SNP, rs3813867 SNP, rs2031920/rs3813867 haplotype and HNSCC risk.

Cao et al. [18] selected 17 case–control studies with 2639 cases and 3450 controls for a meta-analysis of the association between *CYP2E1* rs3813867 and the risk of lung cancer in the Chinese population in 2014, and showed a potential link between the “C” allele carriers of *CYP2E1* rs3813867 and a decreased risk of lung cancer. In our meta-analysis, very limited data were included after our strict selection; thus, no statistical evidence regarding the role of *CYP2E1* rs3813867 in LSCC risk was provided. However, we enrolled five case–control studies [26–28, 33, 39] in our subgroup analysis of “LSCC” for *CYP2E1* rs2031920 and found a negative genetic relationship, which was partly in line with the previous data from LSCC subgroup analysis [47].

The close linkage disequilibrium between rs2031920 and rs3813867 for the *CYP2E1* gene was reported [4–6]. For example, the same genotype frequency distribution was observed in case and control groups of south Indians [14]. However, we observed different genotype frequency distributions between case and control in some other reports [29, 45]. For example, in the Taihang regions of China, the genotype frequency of rs2031920 differs from that of rs3813867 in both the case and control groups [45]. In addition, most case–control studies only measured the single SNP. Thus, we performed a meta-analysis of rs2031920 and rs3813867, respectively; then, we analyzed the role of the rs2031920/rs3813867 haplotype based on the available data. We also conducted an overall and subgroup meta-analysis with four factors (race, SCC type, control source and HWE) under the allele, carrier, heterozygote and dominant models.

To enroll as many eligible case–control studies as possible, a search of five independent online databases (PubMed, Web of Science, Cochrane, Scopus and CNKI) was performed using the overall SCC terms and specific terms, such as ESCC, HNSCC, LSCC and SSCC. Based on our strict criteria, we removed the articles that contained the unconfirmed pathological typing information or failed to provide a genotype frequency distribution in both case and control studies. We observed the absence of large publication bias and the stability of data through Begg’s/Egger’s tests and sensitivity analyses.

Despite this, the shortcomings of the small sample size may still have affected our statistical power. Only one case–control study [38] was included in the “cervical SCC” subgroup analysis of rs2031920 under the allele, carrier, homozygote, heterozygote, and recessive models. Only one case–control study [18] was enrolled in the “lung SCC” subgroup analysis of rs3813867 under all genetic models. Only two studies [25, 36] were enrolled in the “ESCC” subgroup analysis of the rs2031920/rs3813867 haplotype.

In this study, we focused on the genetic role of two polymorphisms within the *CYP2E1* gene in our meta-analysis, and we still cannot rule out the potential genetic effect of other *CYP2E1* polymorphisms (e.g., rs6413432 T/A) and the variant combination between *CYP2E1* and other related genes (e.g., *MDM2*).

For rs3813867, we did not observe obvious heterogeneity in the allele, carrier, homozygote and recessive models, only apart from the heterozygote model. Reduced heterogeneity levels were also observed in the ESCC subgroup analysis compared to the overall analysis. For example, in the allele model, a relatively high heterogeneity level in overall meta-analysis (*P* value of heterogeneity = 0.054, I^2^ = 46.1%) changed to a relatively lower heterogeneity level in the ESCC subgroup (*P* value of heterogeneity = 0.517, I^2^ = 0.0%). A slight reduction was also observed for the heterozygote model (*P* value of heterogeneity from 0.026 to 0.101, I^2^ value from 52.4 to 51.9%), even though significant between-study heterogeneity existed in the ESCC subgroup. We thus performed another meta-analysis, which only enrolled the available case–control studies of ESCC, and similar results were obtained (data not shown).

In addition, we observed remarkable heterogeneity for the allele, carrier, heterozygote, dominant and recessive modes of rs2031920. Even though a stable result was detected in the sensitivity analysis, and no decreased heterogeneity level was observed in the subgroup of ESCC compared with overall meta-analysis. This suggested that mixed factors contributed to the source of heterogeneity of specific ESCC subgroups. We tried to analyze the clinical characterizations, such as gender, age or concomitant pathologies, within the enrolled case–control studies. However, in the ESCC, only six eligible case–control studies were included in the ESCC subgroup, and the adjustment data was very limited for categorization. A larger sample size is required to conduct a more in-depth analysis.

## Conclusions

In conclusion, our meta-analysis data demonstrated that the *CYP2E1* rs2031920 and rs3813867 polymorphisms may be associated with the risk of ESCC. However, this conclusion should be confirmed with more extractable case–control studies.

## Abbreviations

CYP2E1: cytochrome P450 2E1
SCC: squamous cell carcinoma
ESCC: esophageal squamous cell carcinoma
SNP: single nucleotide polymorphisms
HPV: human papillomavirus
MDM2: MDM2 Proto-Oncogene
PRISMA: preferred reporting items for systematic reviews and meta-analyses
CNKI: Chinese National Knowledge Infrastructure
HWE: Hardy–Weinberg equilibrium
NOS: Newcastle–Ottawa Scale
OR: odds ratio
CI: confidence interval
PB: population-based control
HB: hospital-based control
HNSCC: head and neck squamous cell carcinoma
LSCC: lung squamous cell carcinoma
CSCC: cervical squamous cell carcinoma
RFLP: restriction fragment-length polymorphism
SSCP: single-strand conformation polymorphism
